#  “Vaccinating your child during an emergency is more important than ever”: a randomised controlled trial on message framing among Ukrainian refugees in Poland, 2023

**DOI:** 10.2807/1560-7917.ES.2024.29.39.2400159

**Published:** 2024-09-26

**Authors:** Maike Winters, Agnieszka Sochoń-Latuszek, Anastasiia Nurzhynska, Kseniia Yoruk, Katarzyna Kukuła, Mutribjon Bahruddinov, Aleksandra Kusek, Dorota Kleszczewska, Anna Dzielska, Tomasz Maciejewski, Joanna Mazur, Hannah Melchinger, John Kinsman, Piotr Kramarz, Sarah Christie, Saad B Omer

**Affiliations:** 1 European Centre for Disease Prevention and Control (ECDC), Stockholm, Sweden; 2 UNICEF Refugee Response Office in Poland, Warsaw, Poland; 3 Institute of Mother and Child Foundation, Warsaw, Poland; 4 Institute of Mother and Child, Department of Child and Adolescent Health, Warsaw, Poland; 5 Institute of Mother and Child, Clinic of Obstetrics and Gynaecology, Warsaw, Poland; 6 Collegium Medicum, University of Zielona Góra, Department of Humanization of HealthCare and Sexology, Collegium Medicum, Zielona Góra, Poland; 7 Peter O’Donnell Jr. School of Public Health, University of Texas Southwestern, Dallas, USA; 8 Yale School of Public Health, Yale University, New Haven, USA

**Keywords:** vaccination, routine childhood immunisation, vaccine hesitancy, vaccination intention, migrants, refugees, Ukraine, Poland, RCT

## Abstract

**Background:**

Since February 2022, the start of the full-scale war in Ukraine, millions of women and children have fled the country. Vaccination of refugee children is important to protect this vulnerable population from disease.

**Aim:**

We investigate the determinants of vaccination intention in refugee mothers from Ukraine residing in Poland and test the effect of three message frames.

**Methods:**

Participants were randomised into either a control group or one of three intervention groups encouraging vaccination using a specific frame: (i) trust in the Polish health system, (ii) ease of access to vaccination or (iii) risk aversion. Primary outcomes were intention to vaccinate a child in Poland and clicking on a vaccination scheduling link.

**Results:**

The study was completed by 1,910 Ukrainian refugee mothers. Compared with the control group, the risk aversion message significantly increased vaccination intention (adjusted odds ratio (AOR): 2.35, 95% confidence interval (CI): 1.25–4.42) and clicking on the vaccine scheduling link (AOR: 1.53, 95% CI: 1.12–2.09). Messages around trust and ease of access did not have an effect. Important determinants of vaccination intention were perceived importance of vaccination (AOR: 1.12 95% CI: 1.01–1.25) and trusting vaccination information official health institutes (AOR: 1.40 95% CI: 1.06–1.83) and social media (AOR: 2.09 95% CI: 1.33–3.27).

**Discussion:**

Using a risk aversion frame highlighting the vulnerability to infection that refugees face resulted in increased vaccination intention and clicks on a vaccination scheduler. Health workers who interact with Ukrainian refugees could use this frame in their vaccination communication.

**Key public health message**

**What did you want to address in this study and why?**

Millions of women and children have fled Ukraine since the start of the full-scale war. Vaccination coverage among Ukrainian children is relatively low. We investigated what influences vaccination intention among Ukrainian refugee mothers in Poland. We also tested how to best frame a pro-vaccination message to increase vaccination intention and clicking on a link to schedule vaccinations.

**What have we learnt from this study?**

We found that highlighting the vulnerable position Ukrainian refugees are in helped to increase vaccination intention. This also led to more clicks on the vaccine scheduling link, compared with a neutral message. We also found that when mothers perceive vaccination as important, they are more likely get their children vaccinated. Mothers who were likely to return to Ukraine soon, were less likely to get their children vaccinated.

**What are the implications of your findings for public health?**

Our study shows an important way of communicating with Ukrainian refugee mothers about vaccinating their children – by empathising with their difficult and vulnerable situation. The main drivers of vaccination intention can also be leveraged, for instance by emphasising the importance of vaccination to protect their children from infectious diseases.

## Introduction

Since the start of the full-scale war in February 2022 in Ukraine, more than 6 million Ukrainians have fled their country, with another 5 million internally displaced [1]. More than 1.6 million Ukrainian refugees have registered for temporary protection in neighbouring Poland [2]. It is estimated that around 90% of these refugees are women and children [3,4].

Migrants and refugees are especially vulnerable to infectious diseases [5-7]. Difficulty in accessing healthcare and low vaccination coverage in their country of origin, as well as conditions experienced during transit, provide fertile ground for infectious diseases to spread [5,7,8]. According to experts, the most important public health needs related to migrants include a reception system for newly arrived migrants, shelter conditions, screening for infectious diseases, vaccination, follow-up of vaccination, treatment and care, and health education and health promotion [7]. A multitude of barriers often prevent migrants and refugees from accessing healthcare and vaccination services [9]. For instance, there may be a lack of information material in their native language resulting in communication barriers, or a lack of health provider knowledge on vaccination catch up guidelines [10,11]. Furthermore, many vaccines require multiple doses at different time intervals, a schedule which is difficult to follow when migrants and refugees move between countries [12].

In March 2022, a month after the start of the full-scale war in Ukraine, Poland’s health minister issued a decree around the special threat that infectious diseases pose to the public health system [13]. Under this decree, anybody living in Poland for more than 3 months must comply with the Polish mandatory vaccination programme [13]. These vaccines and services are offered free of charge [14].

In Ukraine, confidence in childhood vaccines has been low in recent years with surveys showing only 49% of Ukrainian parents had confidence in them [15] and 50% believed they were effective [16]. A polio outbreak in 2015 and various large measles outbreaks were the consequence of low vaccination rates [17,18]. Shortages of vaccines, underfunding of public services and increasing vaccine scepticism have been named as contributing factors to the low vaccination coverage [17].

From 2016, childhood vaccination coverage in Ukraine increased, reaching 92% for the measles, mumps and rubella (MMR) vaccine and 78% for the polio vaccine in 2019 [19]. Despite this progress, in October 2021, a 17-month-old child was diagnosed with polio [18]. A catch-up vaccination campaign started in February 2022 but was halted due to the war.

Deciding to get vaccinated is a complex behaviour that is influenced by multiple factors. The Behavioural and Social Drivers of Vaccination framework (BeSD) suggests that ‘thinking and feeling’, as well as ‘social processes’ and ‘practical issues’, can be important determinants of vaccination motivation and vaccination behaviour [20]. Thinking and feeling refers to the cognitive and emotional processes around both vaccination and vaccine-preventable diseases [21]. Social processes include vaccination recommendations as well as perceived social norms around vaccination. How pro-vaccine messages are framed may influence social processes, thinking and feeling and the perception of practical issues. This in turn may affect vaccination motivation [21,22].

A recent meta-analysis looking at the effects of behavioural interventions on vaccine uptake found that message framing has, overall, a large positive effect [22]. For instance, one of the trials among mothers of young children ages 0–36 months found that messages that had a gain frame (i.e. focusing on what can be gained by vaccinating the child, such as healthier life or avoidance of disease) or a loss frame (i.e. focusing on increased chances of getting sick if not vaccinated) translated into higher vaccine uptake [23]. The most appropriate type of frame is highly dependent on the context and target audience of the communication campaign [22,24].

Vaccination uptake among Ukrainian refugee children in Poland remains suboptimal in 2023. In order to inform interventions targeted at this population, it is vital to understand the determinants of vaccination intentions, i.e. the intention to have ones’ child vaccinated. Our study aimed to investigate the determinants of vaccination intention and to test the effect of various message frames on the intention of Ukrainian refugee mothers to get their child vaccinated in Poland.

## Methods

### Study design

We conducted an online survey with an embedded randomised controlled experiment. In the experiment, we tested three pro-vaccine messages and compared their effectiveness on increasing the intention to vaccinate a child as well as the likelihood of clicking on a vaccination scheduling link. We calculated that we needed a sample size of 388 participants per group to be able to detect a difference of 10% between intervention and control with 80% power. To account for attrition during the study, we inflated this to a targeted 500 participants per group. The questionnaire was partially based on the published BeSD questionnaire [20], with items on the perceived importance of vaccination, vaccination intentions, social norms, trust in healthcare providers and knowledge around the vaccination schedule. It also contained items that were specific to the Polish and refugee context, such as possession of a vaccination card and likelihood of returning to Ukraine. The full questionnaire is appended in the Supplementary material.

### Recruitment and participants

The study took place online and was targeted at Ukrainian mothers currently residing in Poland and who had at least one child under the age of 7 years. The market research company Rating Online (Kyiv, Ukraine) oversaw the recruitment of participants. Participants were recruited using advertisements on social media and through text messages to phone numbers from mobile phone providers Kyivstar and Vodafone; for data protection reasons, the providers sent the messages; the research team did not have access to the phone numbers. Ukrainians who had left Ukraine and had been residing in Poland after the start of the full-scale war were targeted. Practically, the mobile phone providers could target those who had been in Poland for at least 30 days since the beginning of the year and whose last contact in Poland did not precede their last contact in Ukraine. To further ensure that we reached the target group, screening questions at the start of the survey were used to determine current place of residence, age, nationality, sex, and having at least one child under the age of 7 years. The survey was anonymous.

Before starting the survey, participants read an information sheet that explained the purpose of the study, and they were asked whether they wanted to participate. Only after informed consent was given did the study start. The study was offered in both Ukrainian and Russian languages and the participants selected their preferred language.

### Randomisation and intervention

After completing the first set of questions, participants were randomised 1:1:1:1 into one of four groups: three intervention groups and one control group. Randomisation was achieved using the online survey platform functionality, whereby the platform automatically randomised the participants into the various groups, without the interference of the researchers. The participants were unaware of the randomisation. Three pro-vaccine messages were developed and tested against a control message (Table 1). Briefly, the messages were based on insights from a previous survey among Ukrainian refugee mothers living in Poland, showing a relatively high trust in Polish health workers, a perceived difficulty to access health services in Poland and the tendency to delay childhood vaccination (data not shown (UNICEF 2022)). All intervention messages started with a first sentence that encouraged the mothers to have their children vaccinated in Poland. The next sentence varied per group, which highlighted confidence in Poland’s healthcare system (Group 1: trust), emphasised the accessibility of vaccinations (Group 2: access), or underscored the importance of immunisation to mitigate risks while in a vulnerable state as a migrant (Group 3: risk aversion). At the end of the survey a link to a vaccination scheduling website was provided.

**Table 1:** Messages used in the intervention and control groups to Ukrainian refugee mothers living in Poland, 2023

Group	Message
Control	Dare to be healthy
Intervention 1: trust	Schedule routine vaccinations for your children while you are in Poland! Protect their health and shield them from vaccine-preventable diseases. The Polish health system has a strong immunisation programme that has been successful in preventing serious diseases in children using the highest quality vaccines.
Intervention 2: access	Schedule routine vaccinations for your children while you are in Poland! Protect their health and shield them from preventable diseases. Getting your child vaccinated in Poland is easy and convenient.
Intervention 3: risk aversion	Schedule routine vaccinations for your children while you are in Poland! Protect their health and shield them from preventable diseases. Vaccinating your child during an emergency is more important than ever as your child might be more vulnerable to infectious diseases.

### Outcomes

The randomised experiment had two main outcomes: (i) intention to have a child vaccinated in Poland within the next 6 months, and (ii) clicking on a link to schedule a vaccination.

The first primary outcome was measured by asking both before and after the intervention: ‘Do you intend to vaccinate your child within the next 6 months in Poland?’, to which participants could respond ‘yes’, ‘no’ or ‘not sure’. The second primary outcome was measured as clicking on a link (i.e. click or no click), which was offered at the end of the study with the statement: ‘Click below and make an appointment to get your child vaccinated’.

As a secondary outcome, we measured whether the intervention had an effect on the perceived importance of childhood vaccinations. This was measured both before and after exposure to the intervention or control message with the statement ‘I think it’s important to vaccinate my child’, which participants could agree with on a Likert scale from 0 to 10 (where 0 is strongly disagree and 10 is strongly agree. We also studied the determinants of intention to get vaccinated using the question asked before exposure to the intervention or control message. This analysis was driven by the BeSD model, grouping variables into ‘thinking and feeling’, ‘social processes’ and ‘practical issues’ to understand whether they were associated with intention to vaccinate; the overview of the grouping of the variables is appended in Supplementary Figure S1.

### Statistical analysis

Demographic characteristics of the sample were summarised descriptively. We compared any differences in these characteristics between the assigned groups using chi-square and t-tests. The experiment was analysed using complete case analysis and following the intention-to-treat principle [25]. The first primary outcome (i.e. intention to get a child vaccinated) was dichotomised into ‘yes’ and ‘no/not sure’ and analysed using logistic regression models. In the crude analyses, this was only adjusted for baseline values of the outcome. In the fully adjusted model, we adjusted for the age of the youngest child (0–7 years), level of education of the mother (primary/secondary, vocational, higher education), perceived importance of childhood vaccinations (Likert scale 0–10), perceived social norm to get a child vaccinated (no, yes), knowledge on how to get a child vaccinated in Poland (no, yes), trust in healthcare providers (no trust, somewhat, mostly, fully trust) and the likelihood of returning to Ukraine (no return, return, not sure).

The second primary outcome (i.e. clicking on a vaccine scheduling link), was binary (click or no click) and the effect of the intervention on this outcome was analysed using logistic regression, both crude and adjusted for the same variables as the first primary outcome. The secondary outcome of the experiment (i.e. perceived importance of childhood vaccination) was measured on a Likert scale from 0 to 10. The effect of the interventions on this outcome was analysed using linear regression, which in the crude model was adjusted for the baseline values of this outcome and in the fully adjusted models for the same variables as outlined under the first primary outcome. For both primary outcomes, we conducted a non-registered analysis, comparing the intervention groups to each other.

The analysis of the determinants of intention to get a child vaccinated was informed by the BeSD framework [20,21], whereby we grouped the variables of interest into three blocks: thinking and feeling; social processes; and practical issues. We hypothesised that the three blocks could affect the intention to get a child vaccinated; for the overview of the variables per block we refer to Supplementary Figure S1. The age of the child could, in this model, potentially moderate the associations between the outcome and three variables, namely the importance of vaccination, likelihood of returning to Ukraine and trust in healthcare providers.

The thinking and feeling block contained the education level of the mother (primary/secondary, vocational, higher education), perceived importance of vaccination (scale: 0–10) and likelihood of returning to Ukraine (no return, return, not sure). The social processes block contained perceived social norm of vaccination (no, yes), trust in healthcare providers (no trust, somewhat, mostly, fully), trusted vaccination information sources and the greatest influence on vaccination decisions. For trusted sources, participants were presented with 12 sources to which they could answer ‘no’ or ‘yes’ to indicate which ones they trusted. The sources were: official health institute; television; radio; newspapers; social media; YouTube; other Ukrainian parents in Poland; doctors; family; friends; other; and no one. Only the sources for which more than 5% of the sample answered ‘yes’ were included in the analysis (i.e. official health institutes (74%), social media (7%), other Ukrainian parents (16%), doctors (56%), family (7%), friends (6%)), see also the full list of trust in sources in Supplementary Table S1. For the greatest influence question, a list of 11 sources was presented (family, friends, other parents, religious leaders, co-workers, school, doctors or other health workers, celebrities, public health figures, none), to which participants could answer ‘no’ or ‘yes’ The sources that had more than 5% yes answers were: family (25%), doctors or other health workers (41%), public health figures (20%) and no one (30%). The third and final block, practical issues, contained the variables healthcare seeking behaviour in Poland (I cannot access healthcare, to a non-Ukrainian doctor in Poland, to a Ukrainian doctor in Poland, to Ukraine), Polish language skills (none, low, medium, high), possession of vaccination card (no, yes) and healthcare coverage in Poland (no, yes).

We first analysed the associations between these blocks of variables and the intention to vaccinate a child using logistic regression models. Variables that were statistically significant (p < 0.05) were included in the fully adjusted model. In the fully adjusted logistic regression model we also analysed whether age acted as a moderator for the three variables mentioned above.

All analyses were preregistered on Open Science Framework (https://osf.io/gvwt3) before the data collection was finalised. Data were analysed using StataSE version 17 (StataCorp, College Station, United States).

## Results

Data were collected between 27 June and 18 July 2023. A total of 1,910 Ukrainian mothers living in Poland with at least one child under the age of 7 years completed the study (Figure). In total, 4,748 people entered the study, of which 1,233 did not meet the inclusion criteria. After the first 18 questions of the study, 2,176 mothers were randomised into one of the four groups.

**Figure:**
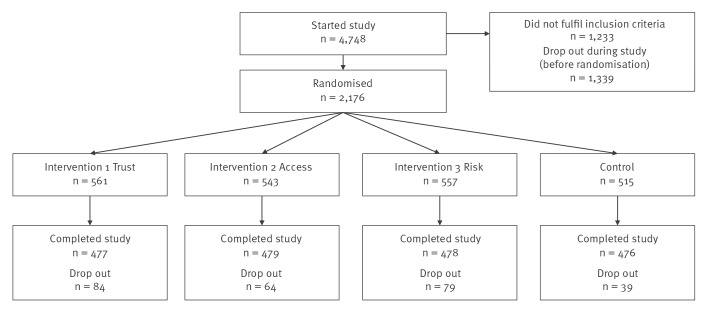
Flowchart of study participants, Ukrainian mothers living in Poland, June 2023–July 2023

About two-thirds of the randomised sample had started or completed higher education and 26% indicated that they would likely not return to Ukraine (Table 2). Most children of the participating mothers were either between 0 and 2 years (37%) or between 5 and 7 years of age (37%).

**Table 2:** Demographics of Ukrainian mothers in each of the four randomised groups, Poland, June 2023–July 2023

Demographics	Group 1: trust n = 561		Group 2: access n = 543		Group 3: risk aversion n = 557		Control n = 515		Total n = 2,176		p value
	n	%	n	%	n	%	n	%	n	%	
Age of child (years)^a^											
0–2	226	40	192	35	208	37	175	34	801	37	0.143
3–4	149	27	150	28	131	24	150	29	580	27	
5–7	186	33	201	37	218	39	190	37	795	37	
Education of mother^a^											
Primary/secondary	89	16	74	14	89	16	68	13	320	15	0.169
Vocational	99	18	118	22	97	17	83	16	397	18	
Higher	373	66	351	65	371	67	364	71	1,459	67	
Mean perceived importance of vaccination (SD)^b^	8.19 (3.04)		7.75 (3.33)		7.98 (3.14)		7.81 (3.29)		7.94 (3.20)		0.098
Likelihood of returning to Ukraine^a^											
Not sure	179	32	200	37	208	37	190	37	777	36	0.031
Not return	133	24	145	27	132	24	146	28	556	26	
Return	249	44	198	36	217	39	179	35	843	39	

SD: standard deviation.

^a^ Analysed using chi-square tests.

^b^ Analysed using ANOVA.

Before exposure to the intervention or control messages, 49% of participants said that they intended to get their child vaccinated in Poland in the next 6 months; see Supplementary Table S2 for the detailed results by group. After reading the intervention message, this increased to 53% for the mothers randomised to the Risk aversion group, which resulted in an adjusted odds ratio (AOR) of 2.35 (95% confidence interval (CI): 1.25–4.42) compared with the control group (Table 3). The message around trust in the Polish health system significantly increased vaccination intentions in the crude analysis (odds ratio (OR): 1.85; 95% CI: 1.01–3.40), but was no longer significant after adjusting (AOR: 1.64; 95% CI: 0.86–3.14). Similarly, the message focusing on ease of access to vaccination had no statistically significant effect on the intention to vaccinate (AOR: 1.35; 95% CI: 0.72–2.54). A post-hoc comparison of the intervention groups (appended in Supplementary Table S3) yielded no differences.

**Table 3:** Primary outcomes for each of the four randomised groups, Ukrainian mothers living in Poland, June 2023–July 2023

Primary outcome	Crude OR		p value	Adjusted OR		p value
	OR	95% CI		AOR	95% CI	
Intention to get a child vaccinated in the next 6 months in Poland						
Control	Reference			Reference		
Trust	1.85	1.01–3.40	0.046	1.64	0.86–3.14	0.133
Access	1.34	0.73–2.47	0.340	1.35	0.72–2.54	0.354
Risk aversion	2.35	1.29–4.28	0.005	2.35	1.24–4.42	0.008
Clicking on vaccination scheduling link						
Control	Reference			Reference		
Trust	1.03	0.77–1.38	0.837	1.07	0.78–1.48	0.661
Access	1.15	0.86–1.54	0.331	1.28	0.94–1.75	0.124
Risk aversion	1.35	1.01–1.79	0.040	1.53	1.12–2.09	0.008

AOR: adjusted odds ratio; CI: confidence interval; OR: odds ratio.

Odds ratios were adjusted for the following variables: age of child; perceived importance of vaccination; social norm; knowledge of vaccination; trust in healthcare providers; likelihood of returning to Ukraine. The outcome ‘intention to vaccinate a child in the next 6 months in Poland’ was also adjusted for the baseline values of this question.

For the second primary outcome, clicking on a vaccination scheduling link, 31% of the participants in the Risk aversion intervention group clicked on the link compared with 25% in the control group (Supplementary Table S4). This translated into an AOR of 1.53 (95% CI: 1.12–2.09), see Table 3. The messages around trust in the Polish health system and ease of access did not translate into an increased clicking rate compared with the control group (Trust AOR: 1.07; 95% CI: 0.78–1.48, Access AOR: 1.28; 95% CI: 0.94–1.75). In the post-hoc comparison between intervention groups, the Risk aversion group was more likely to click on the link compared with the Trust group (AOR: 1.42; 95% CI: 1.04–1.94); for the detailed data we refer to Supplementary Table S5.

In our secondary outcome we looked at the effect of the three intervention messages on the perceived importance of childhood vaccines. Both messages around trust and risk aversion led to higher perceived importance of childhood vaccines compared with the control message. For the trust message, this translated to an increase of 0.23 points on the 10-point importance scale (adjusted coefficient (AC): 0.23; 95% CI: 0.07–0.38). For the risk aversion message, there was an increase of 0.18 points (AC: 0.18; 95% CI: 0.02–0.34), see Table 4.

**Table 4:** Secondary outcome: perceived importance of childhood vaccines, Ukrainian mothers living in Poland, June 2023–July 2023

Assigned group	Crude coefficient		p value	Adjusted^a^ coefficient		p value
	Coefficient	95% CI		Coefficient	95% CI	
Control	Reference		NA	Reference		NA
Trust	0.25	0.09 to 0.42	0.003	0.23	0.07 to 0.38	0.005
Access	0.10	−0.07 to 0.27	0.243	0.06	−0.10 to 0.21	0.462
Risk aversion	0.20	0.03 to 0.37	0.020	0.18	0.02 to 0.34	0.025

CI: confidence interval; NA: not applicable.

^a^ Adjusted for the following variables: age of child; social norm; knowledge of vaccination; trust in healthcare providers.

When looking at the determinants of intention to get a child vaccinated in Poland in the next six months, in the thinking and feeling block, only the perceived importance of vaccination was significantly associated with the intention to get a child vaccinated in Poland (AOR: 1.12; 95% CI: 1.01–1.25), see Table 5 and Supplementary Table S6. For the social processes variables, having some trust, compared with full trust, in healthcare providers was negatively associated with vaccination intention (AOR: 0.37; 95% CI: 0.15–0.90). Trusting vaccination information from social media was associated with increased vaccination intention (AOR: 2.09; 95% CI: 1.33–3.27). Trusting information from official health institutes was similarly positively associated with vaccination intention (AOR: 1.40; 95% CI: 1.06–1.83). Increased age of child had a negative effect on vaccination intention (AOR: 0.66; 95% CI: 0.51–0.85). The age of the child had a moderating effect between the likelihood of returning to Ukraine and vaccination intention (not returning AOR: 0.87; 95% CI: 0.77–0.99). No other moderating effects were observed.

**Table 5:** Determinants of intention to get a child vaccinated in Poland in the next 6 months

Determinants of intention	Adjusted^a^ odds ratio		p value
	AOR	95% CI	
Block 1: thinking and feeling			
**Importance of vaccination**	1.12	1.01–1.25	0.033
**Likelihood of returning to Ukraine**			
Not return	Reference		
Return	0.82	0.43–1.57	0.547
Not sure	0.87	0.44–1.68	0.671
Block 2: social processes			
**Vaccination as social norm**			
No	Reference		
Yes	0.96	0.75–1.23	0.750
**Trust in healthcare providers**			
No trust	0.21	0.04–1.26	0.087
Somewhat	0.37	0.15–0.90	0.029
Mostly	0.93	0.51–1.72	0.828
Fully	Reference		
**Trusted vaccination information sources^b^**			
Official health institute	1.40	1.06–1.83	0.016
Social media	2.09	1.33–3.27	0.001
Doctors	1.08	0.86–1.36	0.494
Block 3: practical issues			
**Polish language**			
None	Reference		
Low	1.43	0.94–2.17	0.096
Medium	1.24	0.81–1.91	0.328
High	1.59	0.85–3.00	0.150
**Possession of vaccination card**			
No	Reference		
Yes	1.29	0.98–1.69	0.071
Moderator			
**Age of child**	0.66	0.51–0.85	0.001
**Age x importance to vaccinate**	1.02	1.00–1.05	0.051
**Age x likelihood to return to Ukraine**			
Not return	Reference		
Return	0.87	0.77–0.99	0.039
Not sure	0.91	0.80–1.03	0.143
**Age x trust in healthcare providers**			
No trust	1.19	0.82–1.73	0.352
Somewhat	1.14	0.95–1.38	0.169
Mostly	0.96	0.85–1.08	0.479
Fully	Reference		

AOR: adjusted odds ratio; CI: confidence interval.

^a^ Adjusted for all other variables in this table.

^b^ Compared to those who did not list these sources as trusted.

## Discussion

In this online, randomised controlled trial, we found that a short message focussing on risk aversion (while also highlighting the vulnerable situation refugees are in) increased vaccination intention and clicks on a vaccination scheduler among refugee mothers from Ukraine currently residing in Poland.

Evidence to date on message framing similarly shows that this can be a low-cost way to increase vaccination intentions [22]. In our study, we found that an absolute increase in vaccination intention of a few percentage points, which on a population level could translate into a meaningful increase in vaccination coverage [23]. Messages can be framed in a number of ways, and our study confirms the importance to robustly test which message frame works in a given situation and with a given population [26]. It can be argued that the message around risk aversion performed well in our study because it acknowledges the difficult situation the refugees are in: feeling seen and being approached with empathy has been shown to work in other settings [27,28]. Furthermore, it may speak to the message being perceived as relevant and timely [24].

While the message that focused on trust in the Polish health system did not have an impact on the two primary outcomes, it did improve the perceived importance of childhood vaccination among the participants. Trust is an important determinant for various health behaviours, including vaccination [29,30]. However, trust in relation to vaccination concerns a range of issues, including the vaccine itself but also extends to health workers and the health system [31]. It may be that messages that address each of these various layers of trust would be more successful in increasing vaccination intention.

The message that highlighted the ease of access to vaccination services did not translate into increased vaccination intention, clicking on the vaccination scheduler, or perceived importance of vaccination. Promoting self-efficacy and response efficacy (i.e. how to get vaccinated) are important parts of vaccination messages [24,32]. It could be that by not providing any further information on where refugees could access vaccination services (apart from providing a link to a vaccination scheduling website) and enhancing self-efficacy, the message was rendered less powerful. Furthermore, it could be that while the messages on trust and ease of access were based on previous insights among this target population, it did not reflect the perceived real-world situation among the participants and thus did not translate into an increase in vaccination intention or clicks.

Trust was an important determinant of vaccination intention – trusting social media and official health institutes to obtain vaccination information was associated with increased intention. Analysis of the determinant factors also suggested that the perceived importance of vaccination and trust in healthcare providers played a role in vaccination intention. Increased age of the child lowered vaccination intention, which may be explained by older children not being due for vaccination. Messages focusing on the current situation refugees are in (such as the risk aversion message) as well as more targeted to mothers of younger children could therefore be helpful in increasing vaccination intention.

Our messages did not have a clear author or ‘messenger’, they were simply presented as blocks of text. It has been shown that the messenger can play a key role in how a message is received [33,34], so it would be important to work with sources who are trusted among refugees from Ukraine, and to co-create pro-vaccine messages with them. Social media, as a trusted source among refugees, could be used for such messaging campaigns, being mindful that such campaigns may not translate into actual vaccination behaviour [35] and that social media can also be a fertile ground for vaccine misinformation [36,37]. Providing trustworthy information through trusted sources is therefore of utmost importance.

Strengths of this study include the possibility to randomise participants, which inherently helps to adjust for (unmeasured) confounders. As a study design, a randomised controlled trial allows for causal interpretation of the results. The large sample size makes generalisation of results more plausible, even though true generalisability is difficult to establish in a highly mobile population with a constantly evolving war situation in their home country. Furthermore, participants who choose to take part in a survey may not be representative of the target population. Especially a topic as sensitive as vaccination may have skewed the sample to mothers who had more extreme attitudes towards childhood immunisation.

Other limitations of our study include the fact that our intervention was presented as a block of text during the study without a clear author. We cannot determine whether the risk aversion message might yield the same results when given by a health worker for instance. Furthermore, it is unclear whether increased vaccination intention among our participants will translate to actual vaccine uptake. Future studies should aim to make the link between messages and actual behaviour. Clicking on a vaccination scheduling link is a proxy behaviour. However, we were not able to follow whether those who clicked on the link made a vaccination appointment and got their children vaccinated. Our primary outcome included a timeframe to increase the relevance of the statement (i.e. intention to get a child vaccinated in the next 6 months). However, this meant that older children of the mothers in our study may not have been due for vaccination in this timeframe, potentially lowering intention to vaccinate. In addition, the control group in this study received a message about health (‘Dare to be healthy’), which could still indirectly be linked to vaccination. This active control may have diluted the results to some extent.

## Conclusion

Our study demonstrates that message framing in vaccination communication can play a role in enhancing vaccination intention. Vaccination is often not the first priority for refugee parents and caregivers, and efforts are needed to encourage them to vaccinate refugee children. Health workers who interact with refugees from Ukraine should empathise with the vulnerable situation the refugees and their children are in, and they may want to consider encouraging them to get vaccinated from that perspective.

## Supplementary Data

519000 Supplementary Material
