# Supplementary material for: “Vaccinating your child during an emergency is more important than ever”: a randomised controlled trial on message framing among Ukrainian refugees in Poland, 2023
Source: Euro Surveill. 2024 Sep 26;29(39):2400159. doi: 10.2807/1560-7917.ES.2024.29.39.2400159 (PMC11484345; doi:10.2807/1560-7917.ES.2024.29.39.2400159)
Supplement: Supplementary Material [file 24-00159_WINTERS_Supplement.pdf]

Supplementary material

"This supplementary material is hosted by *Eurosurveillance* as supporting information alongside the article **“Vaccinating your child during an emergency is more important than ever”: a randomised controlled trial on message framing among Ukrainian refugees in Poland, 2023**, on behalf of the authors, who remain responsible for the accuracy and appropriateness of the content. The same standards for ethics, copyright, attributions and permissions as for the article apply. Supplements are not edited by *Eurosurveillance* and the journal is not responsible for the maintenance of any links or email addresses provided therein."

Supplementary Table S1. Trusted sources of vaccine information

	**Trusted**<br>**N (%)**	**Not trusted**<br>**N (%)**
**Trusted sources of information**		
Official health institutes	1413 (74)	497 (26)
TV	18 (1)	1892 (99)
Radio	6 (0.3)	1904 (99)
Newspapers	5 (0.3)	1905 (99)
Social media	132 (7)	1778 (93)
YouTube	16 (1)	1894 (99)
Other parents from Ukraine living here	311 (16)	1599 (84)
Doctors	1064 (56)	846 (44)
Family	135 (7)	1775 (93)
Friends	110 (6)	1800 (94)
I don’t trust any of them	88 (5)	1822 (95)
**Greatest influence on decision to vaccinate**		
Family	469 (25)	1441 (75)
Friends	44 (2)	1866 (98)
Other parents	83 (4)	1827 (96)

Religious leaders	9 (0.5)	1901 (99)
Coworkers	4 (0.2)	1906 (99)
School	45 (2)	1865 (98)
Doctors or other healthcare workers	790 (41)	1120 (59)
Celebrities or other influencers	9 (0.5)	1901 (99)
Public health figures	379 (20)	1531 (80)
None	571 (30)	1339 (70)

Supplementary Table S2. Intention to get a child vaccinated in Poland in the next 6 months by group

	**Intervention 1**<br>**Trust**<br>**N (%)**	**Intervention 2**<br>**Access**<br>**N (%)**	**Intervention 3**<br>**Risk Aversion**<br>**N (%)**	**Control**<br>**N (%)**	**Overall**<br>**N (%)**
**Baseline**					
No/Not sure	229 (48)	261 (54)	243 (51)	240 (50)	973 (51)
Yes	248 (52)	218 (46)	235 (49)	236 (50)	937 (49)
**Outcome**					
No/Not sure	220 (46)	256 (53)	227 (47)	243 (51)	946 (50)

Yes	257 (54)	223 (47)	251 (53)	233 (49)	964 (50)

Supplementary Table S3. Post-hoc comparison between intervention groups for primary outcome: Intention to get a child vaccinated in Poland in the next 6 months

	Access<br>OR (95% CI)<br>P-value	Trust<br>OR (95% CI)<br>P-value	Risk Aversion<br>OR (95% CI)<br>P-value
Access	-	1.22 (0.65-2.28)<br>0.539	1.74 (0.95-3.19)<br>0.073
Trust	0.82 (0.44-1.54)<br>0.539	-	1.43 (0.77-2.64)<br>0.255
Risk aversion	0.57 (0.31-1.05)<br>0.073	0.70 (0.38-1.29)<br>0.255	-

Supplementary Table S4. Clicking on a vaccination scheduling link by group

	**Intervention 1**<br>**Trust**<br>**N (%)**	**Intervention 2**<br>**Access**<br>**N (%)**	**Intervention 3**<br>**Risk Aversion**<br>**N (%)**	**Control**<br>**N (%)**	**Overall**<br>**N (%)**
**Clicking on link**					
Refuse	356 (75)	347 (72)	331 (69)	358 (75)	1392 (73)
Click	121 (25)	132 (28)	147 (31)	118 (25)	518 (27)

Supplementary Table S5. Post-hoc comparison between intervention groups for primary outcome: Clicking on a vaccination scheduling link

	Access<br>OR (95% CI)<br>P-value	Trust<br>OR (95% CI)<br>P-value	Risk Aversion<br>OR (95% CI)<br>P-value
Access	-	0.84 (0.61-1.15)<br>0.278	1.19 (0.88-1.62)<br>0.257
Trust	1.19 (0.87-1.63)<br>0.278	-	1.42 (1.04 -1.94)<br>0.028
Risk aversion	0.84 (0.62-1.14)<br>0.257	0.70 (0.52-0.96)<br>0.028	-

Supplementary Table S6. Block-wise analysis of determinants of vaccination intentions

	**Adjusted* OR (95% CI)**	**P-value**
**Block 1: Thinking and Feeling**		
**Education mother**		
Primary/Secondary		
Vocational	0.85 (0.60-1.20)	0.354
Reference	0.89 (0.67-1.18)	0.403
**Importance of vaccination**	1.30 (1.25-1.35)	0.000
**Likelihood of returning to Ukraine**		
Not return	Reference	-
Return	0.42 (0.33-0.54)	0.000
Not sure	0.55 (0.43-0.71)	0.000
**Block 2: Social Processes**		
**Vaccination as social norm**		
No	Reference	-
Yes	1.43 (1.16-1.76)	0.001
**Trust in healthcare providers**		
No trust	0.12 (0.05-0.25)	0.000
Somewhat	0.29 (0.21-0.42)	0.000
Mostly	0.59 (0.46-0.75	0.000
Fully	Reference	-
**Trusted vaccination info sources**		
Official health institute	1.45 (1.14-1.85)	0.001
Social media	2.00 (1.33-3.01)	0.001
Other Ukrainian parents in Poland	0.94 (0.71-1.24)	0.646
Doctors	1.36 (1.10-1.69)	0.005
Family	0.85 (0.55-1.32)	0.476
Friends	0.76 (0.47-1.24)	0.274
**Greatest influence on vaccination decision**		
Family	1.08 (0.82-1.40)	0.593
Doctors/other healthcare workers	0.89 (0.69-1.16)	0.390
Public health figures	0.81 (0.62-1.06)	0.125
No one	1.05 (0.77-1.42)	0.772
**Block 3: Practical Issues**		
**Polish language**		
None	Reference	-
Low	1.17 (0.78-1.77)	0.453
Medium	1.06 (0.69-1.62)	0.783
High	1.85 (1.01-3.40)	0.046
**Possession vaccination card**		
No	Reference	-
Yes	1.90 (1.48-2.43)	0.000
**Healthcare coverage in Poland**		
No	Reference	
Yes	1.04 (0.83-1.29)	0.739

**Healthcare seeking**		
I can't access healthcare	Reference	-
To a non-Ukrainian doctor in Poland	1.39 (0.64-3.02)	0.411
To a Ukrainian doctor in Poland	1.10 (0.49-2.48)	0.815
To a doctor in Ukraine	0.54 (0.23-1.29)	0.166
**Moderator**		
**Age of child**	0.74 (0.71-0.77)	0.000

OR = Odds Ratio, CI = Confidence Interval
*Adjusted for the other variables in the same block

Supplementary Figure S1. BeSD-informed framework of potential determinants of intention to vaccinate

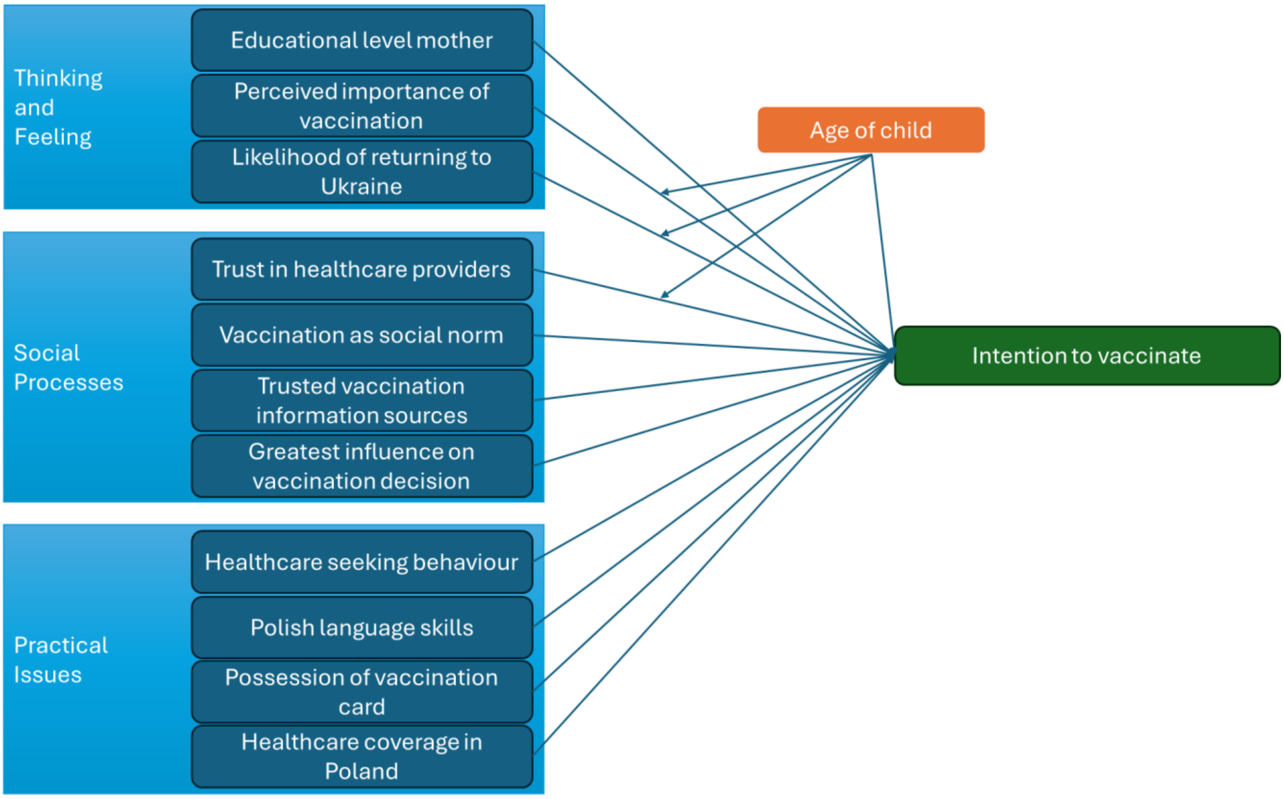

Full survey (in Ukrainian and English)

1. Are you a mother of a child under the age of 7 years?<br><br>1. Yes<br>2. No --> end of survey	1. Чи є у Вас діти віком до 7 років включно?<br><br>1. Так<br><br>2. Ні --> кінець опитування
2. How old is your youngest child?<br><br>1. Less than 1 year old<br>2. 1 year old<br>3. 2 years old<br>4. 3 years old<br>5. 4 years old<br>6. 5 years<br>7. 6 years<br>8. 7 years	2. Скільки років вашій наймолодшій дитині?<br><br>1. Менше 1 року<br>2. 1 рік<br>3. 2 роки<br>4. 3 роки<br>5. 4 роки<br>6. 5 років<br>7. 6 років<br>8. 7 років
3. What is your level of education?<br><br>1. Primary education<br>2. Completed secondary education<br>3. Completed vocational/technical<br>4. Incomplete higher education<br>5. Higher / scientific degree	3. Вкажіть рівень Вашої освіти<br><br>1. Початкова освіта<br>2. Повна середня освіта<br>3. Повна професійно-технічна освіта<br>4. Неповна вища освіта<br>5. Повна вища освіта/ науковий ступінь
4. What is your age? ________________	4. Скільки Вам років? _____________

**5. Do you currently work in Poland?**<br><br>**1. Yes**<br>**2. No**<br>**99. I prefer not to say**	**5. Чи працюєте Ви зараз у Польщі?**<br><br>**1. Так**<br>**2. Ні**<br>**99. Не хочу відповідати**
**6. How would you assess your level of Polish language proficiency?**<br><br>**1. High**<br>**2. Medium**<br>**3. Low**<br>**4. I do not speak Polish at all**<br>**99. I prefer not to say**	**6. Як Ви можете оцінити свій рівень володіння польською мовою?**<br><br>**1. Високий**<br>**[illegible] Середній**<br>**[illegible] Низький**<br>**4. Не володію польською мовою взагалі**<br>**99. Не хочу відповідати**

7. Drop down: List of oblasts	7. В якій області Ви проживали до початку повномасштабної війни 24 лютого 2022 року?
1. Vinnytsia	1. Вінницька
2. Volyn	2. Волинська
3. Dnipropetrovsk	3. Дніпропетровська
4. Donetsk	4. Донецька
5. Zhytomyr	5. Житомирська
6. Zakarpattia	6. Закарпатська
7. Zaporizhzhia	7. Запорізька
8. Ivano-Frankivsk	8. Івано-Франківська
9. Kyiv oblast	9. Київська
10. Kirovohrad	10. Кіровоградська
11. Luhansk	11. Луганська
12. Lviv	12. Львівська
13. Mykolaiv	13. Миколаївська
14. Odesa	14. Одеська
15. Poltava	15. Полтавська
16. Rivne	16. Рівненська
17. Sumy	17. Сумська
18. Ternopil	18. Тернопільська
19. Kharkiv	19. Харківська
20. Kherson	20. Херсонська
21. Khmelnytskyi	21. Хмельницька
22. Cherkasy	22. Черкаська
23. Chernivtsi	23. Чернівецька
24. Chernihiv	24. Чернігівська
25. Kyiv	25. м. Київ

3. Where in Poland do you currently live?<br><br>Drop down: List of provinces	3. Де Ви зараз проживаєте у Польщі? Оберіть воєводство
1. Masovia	1. Мазовецьке (центр Варшава)
2. Lower Silesia	2. Нижньосілезьке (Вроцлав)
3. Kuyavia-Pomerania	3. Куявсько-Поморське (Бидгош, Торунь)
4. Lublin	4. Люблінське (Люблін)
5. Lubusz	5. Любуське (Гожув, Зелена Гура)
6. Łódź	6. Лодзинське (Лодзь)
7. Lesser Poland	7. Малопольське (Краків)
8. Opole	8. Опольське (Ополе)
9. Subcarpathia	9. Підкарпатське (Жешув)
10. Podlasie	10. Підляське (Білосток)
11. Pomerania	11. Поморське (Ґданськ)
12. Świętokrzyskie Voivodeship	12. Свентокшиське (Кельці)
13. Silesia	13. Сілезьке (Катовиці)
14. Greater Poland	14. Великопольське (Познань)
15. Warmia-Masuria	15. Вармінсько-Мазурське (Ольштин)
16. West Pomerania	16. Західнопоморське (Щецин)

BeSD Question (adapted)<br><br>9. I think it's important to vaccinate my child<br><br>0: I strongly disagree<br>1<br>2<br>3<br>4<br>5<br>6<br>7<br>8<br>9<br>10: I strongly agree	9. Чи погоджуєтеся Ви з твердженням про те, що вакцинувати свою дитину важливо?<br><br>0: Зовсім не погоджуюся<br>1<br>2<br>3<br>4<br>5<br>6<br>7<br>8<br>9<br>10: Повністю погоджуюся
BeSD Question<br><br>10. Do you think most of your close family and friends want you to get your child vaccinated?<br><br>1. Yes<br>2. No<br><br>99. Difficult to answer/Not sure	10. Як ви думаєте, більшість вашої родини та друзів хочуть, щоб Ваша дитина була вакцинована?<br><br>1. Так<br>2. Ні<br><br>99. Важко відповісти/Не впевнена
BeSD Question<br><br>11. Do you know how to get your child vaccinated in Poland?<br><br>1. Yes<br>2. No	11. Чи знаєте ви, як зробити щеплення своїй дитині в Польщі?<br><br>1. Так<br>2. Ні

BeSD Question<br><br>12. Poland has a schedule of vaccines for children. As far as you know, has your child received none, some, or all of these vaccines?<br><br>1. None<br>2. Some<br>3. All<br><br>99. Difficult to answer/Not sure	12. У Польщі є календар вакцинації дітей. Чи отримувала ваша дитина щеплення згідно цього календаря<br><br>1. Ні, не отримувала жодних щеплень<br><br>2. Отримувала деякі щеплення<br><br>3. Отримала усі щеплення<br><br>99. Важко відповісти/Не впевнена
13. If some or none (if 12=1 or 2: what was the reason for your decision not to vaccinate your child? MULTIPLE RESPONSE<br><br>1. I'm concerned about the safety and side effects of vaccines<br>2. I don't have easy access to healthcare services in Poland<br>3. I don't know the vaccine schedule/requirements in Poland<br>4. I don't know where to get vaccinated in Poland<br>5. I don't trust the healthcare system and/or medical authorities in Poland<br>6. I don't believe my child needs to be vaccinated (don't believe on the threat of the diseases)<br>7. I was advised by health provider not to receive the vaccines (contraindications, etc)<br>8. I refuse to vaccinate my child due to my religious beliefs<br>9. I prefer to vaccinate my child when back to Ukraine<br>97. Other (please, specify)<br><br>99. Difficult to answer/Not sure	13. Якщо дитина отримала деякі щеплення або жодних (якщо 12=1 або 2). Яка причина вашого рішення не вакцинувати Вашу дитину? МОЖЛИВО ДЕКІЛЬКА ВІДПОДІДЕЙ<br><br>1. Я стурбована (-ний) безпечністю та побічними ефектами від вакцин<br><br>2. Я не маю доступу до медичних послуг у Польщі<br><br>3. Я не знаю графіку вакцинації/вимог в Польщі<br><br>4. Я не знаю, де зробити щеплення в Польщі<br><br>5. Я не довіряю системі охорони здоров'я та/або медичним органам у Польщі<br><br>6. Я не думаю, що моїй дитині потрібно робити щеплення (не вірю в загрозу захворювань)<br><br>7. Лікар порадив мені не робити щеплення (протипоказання тощо)<br><br>8. Я відмовляюся робити щеплення своїй дитині через свої релігійні переконання<br><br>9. Я вважаю за краще вакцинувати свою дитину, коли повернуся в Україну<br><br>97. Інше (вкажіть)______<br><br>99. Важко відповісти/Не впевнена

BeSD Question<br><br>14. How much do you trust the healthcare providers who vaccinate children?<br><br>1. Do not trust<br>2. Somewhat trust<br>3. Mostly trust<br>4. Fully trust<br><br>99. Difficult to answer/Not sure	14. Наскільки ви довіряєте медичним працівникам, які проводять вакцинацію дітей?<br><br>1. Зовсім не довіряю<br><br>2. Не дуже довіряю<br><br>3. Переважно довіряю<br><br>4. Повністю довіряю<br><br>99. Важко відповісти/Не впевнена
15. What should vaccine providers do to better address your concerns, if anything? UP TO TWO ANSWERS<br><br>1. Provide the information in my language<br>2. Spend more time during the consultations<br>3. Review my child vaccine records<br>97. Other (specify)<br><br>99. Difficult to answer/Not sure	15. Що з переліченого могло б покращити роботу медичних працівників, які вакцинують дітей? ОБЕРІТЬ ДО ДВОХ ВІДПОВІДЕЙ<br><br>1. Надання інформації рідною для мене мовою<br><br>2. Збільшення часу консультації<br><br>3. Врахування попередніх щеплень моєї дитини<br><br>97. Інше (вкажіть) ________<br><br>99. Важко відповісти
16. Do you have a vaccination passport (certificate) or any vaccination records of your children, and did you take it with you when leaving Ukraine?<br><br>1. Yes, I have it (a copy or actual record)<br>2. No, I don't have it (and I can't access it)<br>99. I prefer not to answer	16. Чи є у вас паспорт (довідка) вакцинації або будь-які записи про щеплення Ваших дітей, і чи брали Ви їх, виїжджаючи з України?<br><br>1. Так, маю (копія або оригінальний документ)<br><br>2. Ні, не маю (і не можу отримати до них доступ)<br>99. Не хочу відповідати

**17. Did you have any issues with understanding or translating your vaccine records?**<br><br>**1. Yes**<br>**2. No**<br>**99. I don't know**	**17. Чи виникали у вас проблеми з розумінням або перекладом ваших записів про щеплення?**<br><br>**1. Так**<br>**2. Ні**<br>**99. Важко відповісти**
**18. Do you have any kind of health care coverage in Poland (health insurance, private health insurance, etc)?**<br><br>**1. Yes**<br>**2. No**<br>**99. I don't know**	**18. Чи маєте ви медичну страховку в Польщі (державна або приватна)?**<br><br>**1. Так**<br>**2. Ні**<br>**99. Важко відповісти**
**19. Have you encountered barriers when you tried to access vaccination services in Poland?**<br><br>**1. I haven't tried to access**<br>**2. No, we did not face any difficulties**<br>**3. Yes (please, describe) ________**	**19. Чи стикалися ви з будь-якими труднощами, коли намагались зробити щеплення у Польщі?**<br><br>**1. Не намагались зробити щеплення**<br>**2. Ні, не стикались з трудношами**<br>**3. Так (опишіть, які саме проблеми) ________**
**BeSD Question (adapted)**<br><br>**20. Do you intend to vaccinate your child in the next 6 months in Poland?**<br><br>**1. Yes**<br>**2. No**<br>**99. Difficult to answer/Not sure**	**20. Чи маєте Ви намір вакцинувати свою дитину протягом наступних 6 місяців у Польщі?**<br><br>**1. Так**<br>**2. Ні**<br>**99. Важко відповісти/Не впевнена**

**21. Which source of vaccine information do you trust the most? MULTIPLE RESPONSE**<br><br>**1. Official health institutes**<br>**2. TV**<br>**3. Radio**<br>**4. Newspapers**<br>**5. Social media**<br>**6. YouTube**<br>**7. Other parents from Ukraine living here**<br>**8. Doctors**<br>**9. Family**<br>**10. Friends**<br>**97. Other (specify)**<br>**98. I don't trust any of them**<br>**99. Difficult to answer / I prefer not to say**	**21. Яким джерелам інформації про вакцинацію ви довіряєте найбільше? МОЖЛИВО ДЕКІЛЬКА ВІДПОДІДЕЙ**<br><br>**1. Офіційні медичні установи**<br>**2. Телебачення**<br>**3. Радіо**<br>**4. Газети**<br>**5. Соціальні мережі**<br>**6. YouTube**<br>**7. Батьки інших дітей, що живуть у Польщі**<br>**8. Лікарі**<br>**9. Родина**<br>**10. Друзі**<br>**97. Інше (вкажіть) ________**<br>**98. Жодному не довіряю**<br>**99. Важко відповісти / Не хочу відповідати**
**22. Which of the following has the greatest influence on your decision on whether to vaccinate your child, or NOT? MULTIPLE RESPONSE**<br><br>**1. Family**<br>**2. Friends**<br>**3. Other parents**<br>**4. Religious leaders**<br>**5. Coworkers**<br>**6. School**<br>**7. Doctors or other healthcare workers**<br>**8. Celebrities or other influencers**<br>**9. Public health figures**<br>**97.Other (specify)**<br>**98. None**<br>**99. Difficult to answer/Not sure**	**22. Хто з переліку найбільше впливає на ваше рішення вакцинувати або не вакцинувати дитину?**<br><br>**МОЖЛИВО ДЕКІЛЬКА ВІДПОДІДЕЙ**<br>**1. Родина/сім’я**<br>**2. Друзі**<br>**3. Батьки інших дітей, що живуть у Польщі**<br>**4. Релігійні лідери**<br>**5. Колеги по роботі**<br>**6. Школа**<br>**7. Лікарі або інші медичні працівники**<br>**8. Знаменитості або інші впливові особистості**<br>**9. Уповноважені працівники установ з охорони здоров’я**<br>**97. Інше (вкажіть) ________**<br>**98. Ніхто з перелічених**<br>**99. Важко відповісти/Не впевнена**

**23. When your child needs medical care, where do you go?**<br><br>1. To a non-Ukrainian doctor in Poland<br>2. To a Ukrainian doctor in Poland<br>3. To a doctor in Ukraine<br>4. I can’t access health care<br>99. I don’t know	**23. Коли вашій дитині потрібна медична допомога, куди Ви звертаєтесь?**<br><br>1. До польського лікаря у Польщі<br>2. До українського лікаря у Польщі<br>3. До лікаря в Україні<br>4. Не маю доступу до медичних послуг<br>99. Важко відповісти
**24. Which of the following best describes your financial situation?**<br><br>1. Not enough<br>2. Enough, but only for everyday expenses<br>3. Mostly enough<br>4. Enough for almost everything<br>5. More than enough<br>99. I prefer not to answer	**24. Що з наведеного найкраще описує ваше фінансове становище?**<br><br>1. Недостатньо коштів<br>2. Достатньо лише на повсякденні витрати<br>3. В основному достатньо<br>4. Достатньо на майже все, що потрібно<br>5. Більш ніж достатньо<br>99. Не хочу відповідати
**25. How likely are you to return to Ukraine permanently?**<br><br>1. I will return to Ukraine as soon as possible<br>2. I'll probably return as soon as possible<br>3. I probably WON'T return, even if it's possible<br>4. I will not return in any case<br>99. Difficult to answer/Not sure	**25. Чи плануєте Ви повертатися в Україну на постійне проживання?**<br><br>1. Однозначно повернуся в найближчому часі<br>2. Можливо повернуся в найближчому часі<br>3. Скоріше НЕ повернуся, навіть якщо це буде можливо<br>4. НЕ планую повертатися взагалі<br>99. Важко відповісти/Не впевнена

**26. Do you have a PESEL number?**<br><br>**1. Yes**<br>**2. No**<br>**99. I prefer not to answer**	**26. У вас є номер PESEL?**<br><br>**1. Так**<br><br>**2. Ні**<br><br>**99. Важко відповісти**
**27. INTERVENTION (one of the for messages below was presented)**	**27. ІНТЕРВЕНЦІЯ**
**27.1_TRUST Read the following:**<br><br>*Schedule routine vaccinations for your children while you are in Poland! Protect their health and shield them from vaccine preventable diseases.*<br><br>*Polish health system has a strong immunization program, that has been successful in preventing serious diseases in children using the highest quality vaccines.*<br><br>**GO TO THE NEXT QUESTION**	**27.1_TRUST Прочитайте наступне:**<br><br>**Запишіть своїх дітей на планові щеплення, поки ви перебуваєте у Польщі! Захистіть їх здоров'я та збережіть від захворювань, яким можна запобігти за допомогою вакцин.**<br><br>**Система охорони здоров'я в Польщі має потужну програму імунізації, яка успішно запобігає серйозним захворюванням у дітей за допомогою найякісніших вакцин.**<br><br>**ПЕРЕЙДІТЬ ДО НАСТУПНОГО ПИТАННЯ**
**27.2_ACCESS Read the following:**<br><br>*Schedule routine vaccinations for your children while you are in Poland! Protect their health and shield them from preventable diseases.*<br><br>*Getting your child vaccinated in Poland is easy and convenient.*<br><br>**GO TO THE NEXT QUESTION**	**27.2_ACCESS Прочитайте наступне:**<br><br>**Запишіть своїх дітей на планові щеплення, поки ви перебуваєте в Польщі! Захистіть їх здоров'я та збережіть від захворювань, яким можна запобігти за допомогою вакцин.**<br><br>**Зробити щеплення дитині в Польщі – це легко і зручно.**

	ПЕРЕЙДІТЬ ДО НАСТУПНОГО ПИТАННЯ
27.3_RISK **Read the following:**<br><br>*Schedule routine vaccinations for your children while you are in Poland! Protect their health and shield them from preventable diseases.*<br><br>*Vaccinating your child during emergency is more important than ever as your child might be more vulnerable to infections.*<br><br>GO TO THE NEXT QUESTION	27.3_RISK **Прочитайте наступне:**<br><br>**Запишіть своїх дітей на планові щеплення, поки ви перебуваєте в Польщі! Захистіть їх здоров'я та збережіть від захворювань, яким можна запобігти за допомогою вакцин.**<br><br>**Вакцинація вашої дитини під час війни важливіша, ніж будь-коли, оскільки в цей час діти можуть бути більш вразливі до інфекцій.**<br><br>**ПЕРЕЙДІТЬ ДО НАСТУПНОГО ПИТАННЯ**
27.4_CONTROL **Read the following:**<br><br>*Dare to be healthy.*<br><br>GO TO THE NEXT QUESTION	27.4_CONTROL **Прочитайте наступне:**<br><br>**Наважтеся бути здоровими.**<br><br>**ПЕРЕЙДІТЬ ДО НАСТУПНОГО ПИТАННЯ**

BeSD Question (adapted)<br><br>28. I think it's important to vaccinate my child<br><br>0: I strongly disagree<br>1<br>2<br>3<br>4<br>5<br>6<br>7<br>8<br>9<br>10: I strongly agree	28. Чи погоджуєтеся Ви з тим, що вакцинувати свою дитину важливо?<br><br>0: Зовсім не погоджуюся<br>1<br>2<br>3<br>4<br>5<br>6<br>7<br>8<br>9<br>10: Повністю погоджуюся
29. Do you intend to vaccinate your child in the next 6 months in Poland?<br><br>1. Yes<br>2. No<br><br>99. Difficult to answer/Not sure	29. Чи маєте Ви намір вакцинувати свою дитину протягом наступних 6 місяців у Польщі?<br><br>1. Так<br>2. Ні<br>99. Важко відповісти/Не впевнена
30. Do you think most of your close family and friends want you to get your child vaccinated?<br><br>1. No<br>2. Yes<br><br>99. Difficult to answer/Not sure	30. Як ви думаєте, більшість вашої родини та друзів хочуть, щоб Ваша дитина була вакцинована?<br><br>1. Так<br>2. Ні<br>99. Важко відповісти/Не впевнена

**31. Behavioral outcome:**<br><br>***Click below and make an appointment to get your child vaccinated.***<br><br>***CLICK***	**31. Поведінковий експеримент:**<br><br>**Натисніть на посилання нижче та зареєструйтеся на прийом для вакцинації вашої дитини.**<br><br>**ЗАПИС НА ПРИЙОМ ДО ЛІКАРЯ**
